# Effects of Listening to Preferred versus Non-Preferred Music on Repeated Wingate Anaerobic Test Performance

**DOI:** 10.3390/sports7080185

**Published:** 2019-07-29

**Authors:** Christopher G. Ballmann, Daniel J. Maynard, Zachary N. Lafoon, Mallory R. Marshall, Tyler D. Williams, Rebecca R. Rogers

**Affiliations:** Department of Kinesiology, Samford University, Birmingham, AL 35229, USA

**Keywords:** Wingate, anaerobic power, anaerobic capacity, music preference

## Abstract

The purpose of this study was to examine the effects of listening to preferred or non-preferred music on repeated sprint performance. Fourteen physically active males (ages 18–25 years) were recruited for this study. In a counterbalanced crossover study design, participants completed two separate visits. During each visit, participants listened to either preferred or non-preferred music and completed 3 × 15 s Wingate Anaerobic Tests (WAnTs) separated by 2 min active recovery periods. Each visit was separated by a minimal recovery period of 48 h. Anaerobic performance measures, heart rate, rate of perceived exertion (RPE), and motivation were analyzed. Mean power (*p* = 0.846, effect size (ES) = 0.019), anaerobic capacity (*p* = 0.686, ES = 0.058), and total work (*p* = 0.677, ES = 0.039) were not significantly different between preferred and non-preferred music conditions. Mean heart rate (*p* = 0.608; ES = 0.125) was also unchanged. Motivation to exercise (*p* < 0.001; ES = 1.520) was significantly higher in the preferred music condition. Additionally, the rate of perceived exertion (RPE) (*p* = 0.028; ES = 0.540) was significantly lower during the preferred music condition. Our results show that listening to preferred music showed no ergogenic benefit during repeated anaerobic cycling sprints when compared to non-preferred music. However, preferred music increased motivation to exercise and decreased perceived exertion. The results from this study could hold important implications for the application of music and enduring repeated high-intensity sprint exercise.

## 1. Introduction

Music is an external source that serves as an ergogenic aid in a wide arrange of exercise modes and intensities [1,2,3]. Although the exact mechanisms for improvements in performance while listening to music are debated, much of the previous evidence suggests that music dissociates focus from exertion during exercise. Indeed, ratings of perceived exertion (RPE) during exercise have been shown to decrease with music by multiple groups [4,5]. Additionally, other evidence has shown changes in mood, motivation, heat rate, and arousal, which may also cause increases in performance [1,6,7]. Music preference has shown to be an important factor in determining the ergogenic potential of music [1,2]. However, how preference affects previously mentioned mechanisms is unclear, especially in anaerobic exercise.

Music and anaerobic exercise has largely been studied using pre-determined music and has revealed mixed results [8,9,10,11,12]. Brooks et al. reported increases in peak and mean power during 2 × 30 s Wingate Anaerobic Tests (WAnTs) when comparing music to no-music conditions [9]. Simpson et al. reported decreased time to completion during a 400 m sprint using music versus no music [12]. However, other studies have shown no effect of music on anaerobic performance. Rad et al. showed no ergogenic benefits of music during a 100 m swim sprint [13]. Further supporting this, Atan et al. showed that varying tempos (i.e., fast versus slow) of music had no impact on power measurements during running and cycle-based power assessments when compared to no music [8]. Almost all previous evidence investigating music and anaerobic performance has used pre-determined music without controlling for music preference. Thus, the disparities among previous findings may be due to individual differences in music preference, leaving the need for further study.

Music preference has been shown by multiple groups to play a key role in determining the ergogenic potential of music [1,2,14]. Listening to preferred music has been shown to be more stimulating when compared to non-preferred music, causing increases in heart rate and feelings of energy [15]. Dyrlund et al. reported that participants listening to their preferred music reported higher levels of disassociation from exercise, albeit exercise enjoyment was unchanged [14]. Nakamura et al. showed increased endurance cycling performance and decreased RPE when individuals listened to preferred music versus non-preferred music [2]. Further supporting the influence of preference on performance, Ballmann et al. showed increases in repetitions performed, power, and velocity during a single resistance-training bout [1]. Of particular note, motivation to exercise was also significantly higher in the preferred music condition versus the non-preferred. While the efficacy of listening to preferred music has been established in endurance and resistance exercise, it is less clear how preferred music impacts anaerobic sprint performance.

While the previous evidence on music preference and exercise performance is intriguing, there are still factors that should be addressed through future research. All of the previous studies on preferred versus non-preferred music and exercise performance have only investigated one single exercise bout. Thus, it is currently unknown how music preference impacts repeated exercise performance. In addition, all of the previous preference investigations did not control for volume of music. Previous evidence has shown that music volume may impact exercise performance [16]. Lastly, no music preference investigations to date have investigated repeated anaerobic sprint performance. Thus, the purpose of the following study was to investigate the effect of listening to preferred versus non-preferred music on repeated anaerobic cycling sprint performance, RPE, heart rate, and motivation to exercise. We hypothesized that listening to preferred music would increase anaerobic performance, heart rate, and motivation in addition to decreasing RPE when compared to non-preferred music.

## 2. Materials and Methods

### 2.1. Participants

Fourteen physically active male participants (age (years) = 20.14 ± 1.79, height (cm) = 179.57 ± 7.11, body mass (kgs) = 78.2 ± 14.09) were recruited from the Birmingham, Alabama area to participate in this study. Accruing at least 150 min/week of moderate-intensity physical activity was defined as physically active [17]. To determine suitability for exercise, all participants completed a physical activity readiness questionnaire (PAR-Q). All participants were free from lower body injury for 6 months prior to participation. Before each visit, participants were asked to refrain from vigorous activity 24 h prior and from consuming caffeine, nicotine, and alcohol 12 h prior. Written and informed consent was obtained from each participant prior to any data collection. The study was conducted in accordance with the Declaration of Helsinki, and the Samford University Institutional Review Board (IRB) approved the protocol.

### 2.2. Preferred and Non-preferred Music Selection

Preferred (PREF) and non-preferred (NON-PREF) music were selected as previously described by Ballmann et al. [1]. Participants were given a music preference survey with six popular music genres to choose from. These genres included rhythm and blues, country, rock and roll/hard rock, rap/hip hop, pop, and dance/electronic. For each genre, a list of 5 songs from the Billboard Top 10 singles of 2018 were provided. Participants were asked to rank their favorite to least favorite genre. The favorite genre was used or the PREF trial and least favorite for the NON-PREF trial. Participants were asked to pick their favorite song out of the PREF genre. For the NON-PREF, a random song was chosen out of the NON-PREF genre. For the PREF genre selection, six participants chose rock and roll/hard rock, six chose rap/hip hop, and two chose pop. For the NON-PREF genre selection, ten participants chose country, two chose rap/hip hop, and two chose pop. The mean beats per minute for the preferred song selection was 127 beats/min^−1^ ± 28, while the mean beats per minute for the NON-PREF song selection was 128 beats/min^−1^ ± 31. The same electronic device and pair of headphones were used to play all music and the device was set to the same volume level for all participants.

### 2.3. Procedures

After music preference was documented, participant height and weight were recorded and a heart rate monitor (Polar H7, Polar Electro, Bethpage, NY, USA) was placed on each participant. Participants then completed a series of 3 × 15 s repeated Wingate Anaerobic Tests (WAnTs) on an electronically braked cycle ergometer (Velotron, Racermate Inc., Seattle, WA, USA) [18]. Seat height was situated to where, with the crank at the bottom and the foot secured to the pedal with toe straps, the knee had about 5 degrees of flexion [19]. Seat height was adjusted for each participant and recorded for the following trial. Participants began by warming-up for 5 min at an unloaded resistance. Resistance for the test was calculated using 7.5% of the participant’s body mass. Following the warm-up, the corresponding music was started and there was a 10 s countdown phase for participants to reach maximum pedaling rate [19]. The load was then immediately added, and participants pedaled as fast as possible for 15 s. This was repeated 2 more times with 2 min active recovery rest periods in which participants pedaled at their own pace against unloaded resistance. Music was not played during the recovery periods. Visual feedback and verbal encouragement were not provided. After successful completion of each WAnT, performance variables, heart rate, rate of perceived exertion (RPE; Scale 1–10), and motivation were obtained. Motivation was obtained using a visual analog scale with a 100 mm line [1]. Ranging from most motivated to least motivated ever, participants marked how motivated they felt to exercise during the WAnT. Scores were obtained by measuring from zero to the point that was marked on the 100 mm line. Each visit was separated by a minimal recovery period of 48 h. All anaerobic performance measures were calculated over each 15 s WAnT period via Velotron Software (v4 1.0.6 Velotron, Racermate Inc., Seattle, WA, USA).

### 2.4. Statistical Analyses

All data was analyzed using SPSS 25 (IBM, Armonk, NY, USA). For test to test difference, a 2 × 3 (Condition × Test) repeated measures ANOVA was used with Bonferroni corrections for multiple comparisons if warranted. A paired samples t-test was used to analyze average outcomes over all three WAnTs. Effect sizes (ES) were calculated using Cohen’s d effect size calculator for t-test and interpreted as: 0.2—small; 0.5—moderate; 0.8—large [20,21]. All data are presented as mean ± standard deviation (SD). Significance was set at *p* ≤ 0.05 a priori.

## 3. Results

### 3.1. Anaerobic Performance Measures

**Figure 1:**
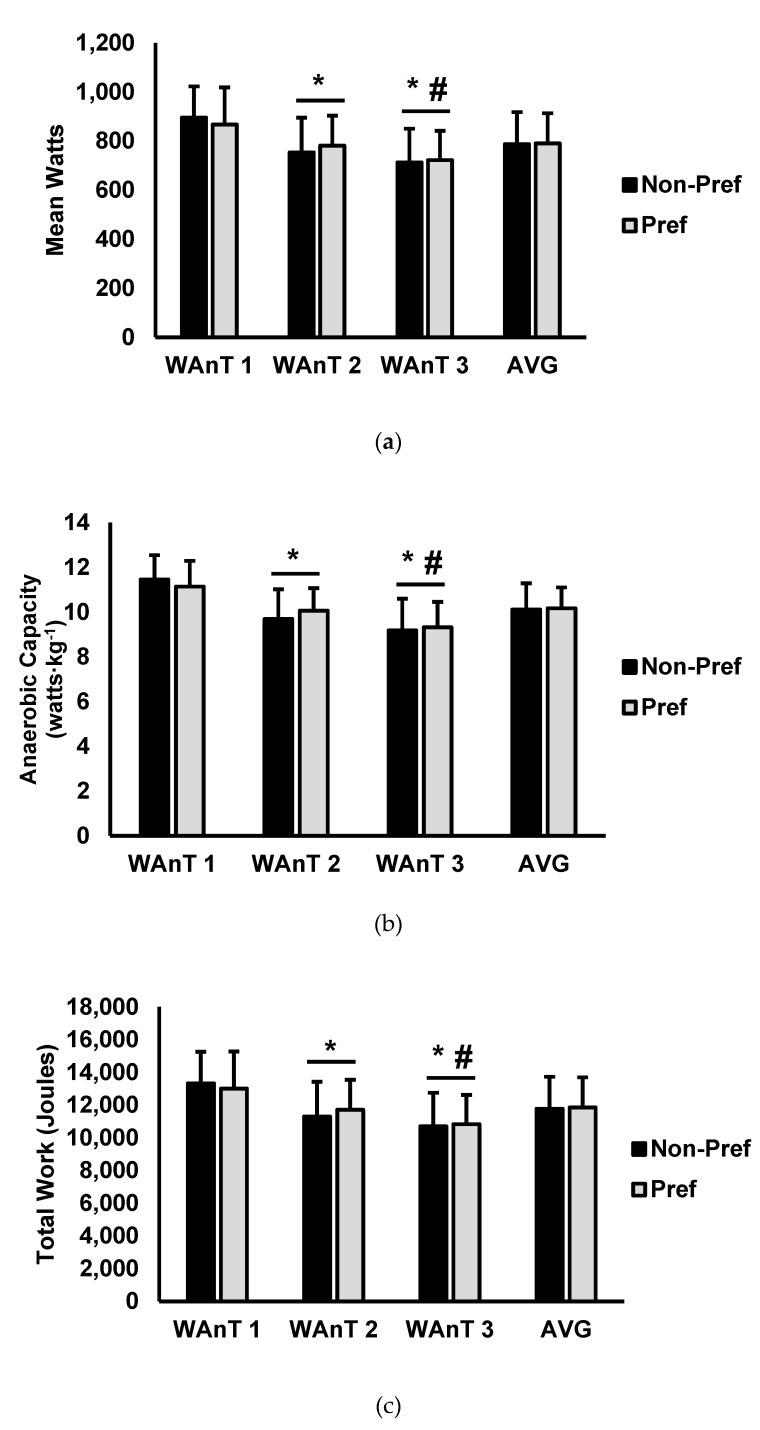
(**a**) Mean power output (watts), (**b**) anaerobic capacity (watts∙kg^−1^), (**c**) total work (joules) for WAnT1, WAnT2, WAnT3, and the average (AVG) over all three. Data are presented as mean ± SD. * indicates significantly different from WAnT1. # indicates significantly different from WAnT2.

Test-to-test performance and average performance over the entire three WAnTs are presented in Figure 1. For mean power (watts) Figure 1a, there was a main effect for test (*p* < 0.001; η_p_^2^ = 0.783) while there was no main effect for condition (*p* = 0.958; η_p_^2^ = 0.001) or interaction for condition and test (*p* = 0.121; η_p_^2^ = 0.047). Analysis of multiple comparisons for test revealed that the first test (PREF = 867.2 watts ± 151.1, NON-PREF = 896.1 watts ± 126.2) was significantly higher than both second (PREF = 781.3 watts ± 122.0, NON-PREF = 754.0 watts ± 140.8; *p* < 0.001) and third WAnTs (PREF = 722.5 watts ± 119.0, NON-PREF = 713.3 watts ± 136.9; *p* < 0.001). In addition, the mean power in the third WAnT was significantly lower than in the second WAnT (*p* < 0.001). Average mean watts was not significantly altered by music preference (NON-PREF = 790.1 watts ± 122.7, PREF = 787.8 watts ± 129.7; *p* = 0.846, ES = 0.019). 

For anaerobic capacity (mean power/kg body mass) (Figure 1b), there was a main effect for test (*p* < 0.001; η_p_^2^ = 0.771), while there was no main effect for condition (*p* = 0.878; η_p_^2^ < 0.001) or interaction for condition and test (*p* = 0.148; η_p_^2^ = 0.068). Analysis of multiple comparisons for test revealed that the first test (PREF = 11.1 watts∙kg^−1^ ± 1.1, NON-PREF = 11.4 watts∙kg^−1^ ± 1.0) was significantly higher than both second (PREF = 10.0 watts∙kg^−1^ ± 1.0, NON-PREF = 9.6 watts∙kg^−1^ ± 1.3; *p* < 0.001) and third WAnTs (PREF = 9.3 watts∙kg^−1^ ± 1.1, NON-PREF = 9.1 watts∙kg^−1^ ± 1.4; *p* < 0.001). The anaerobic capacity in the third WAnT was significantly lower than the second WAnT (*p* < 0.001). The average anaerobic capacity was not significantly different between music conditions (NON-PREF = 790.1 watts∙kg^−1^ ± 122.7, PREF = 787.8 watts∙kg^−1^ ± 129.7; *p* = 0.686, ES = 0.058). 

For total work (joules) (Figure 1c), there was a main effect for test (*p* < 0.001; η_p_^2^ = 0.764), while there was no main effect for condition (*p* = 0.918; η_p_^2^ < 0.001) or interaction for condition and test (*p* = 0.155; η_p_^2^ = 0.138). Similar to the other performance outcomes, analysis of multiple comparisons for test revealed that the first test (PREF = 13,009 joules ± 2266, NON-PREF= 13,334 joules ± 1923) was significantly higher than both second (PREF = 17,172 joules ± 1837, NON-PREF = 113,089 joules ± 2,111; *p* < 0.001) and third WAnTs (PREF = 10,836 joules ± 1785, NON-PREF = 10,699 joules ± 2052; *p* < 0.001). Total work in the third WAnT was significantly lower than the second WAnT (*p* < 0.001). Average total work was not significantly different between music conditions (NON-PREF = 11,780 joules ± 123, PREF = 11,855 joules ± 1841; *p* = 0.677; ES = 0.039).

### 3.2. Heart Rate, Motivation to Exercise, RPE

**Figure 2:**
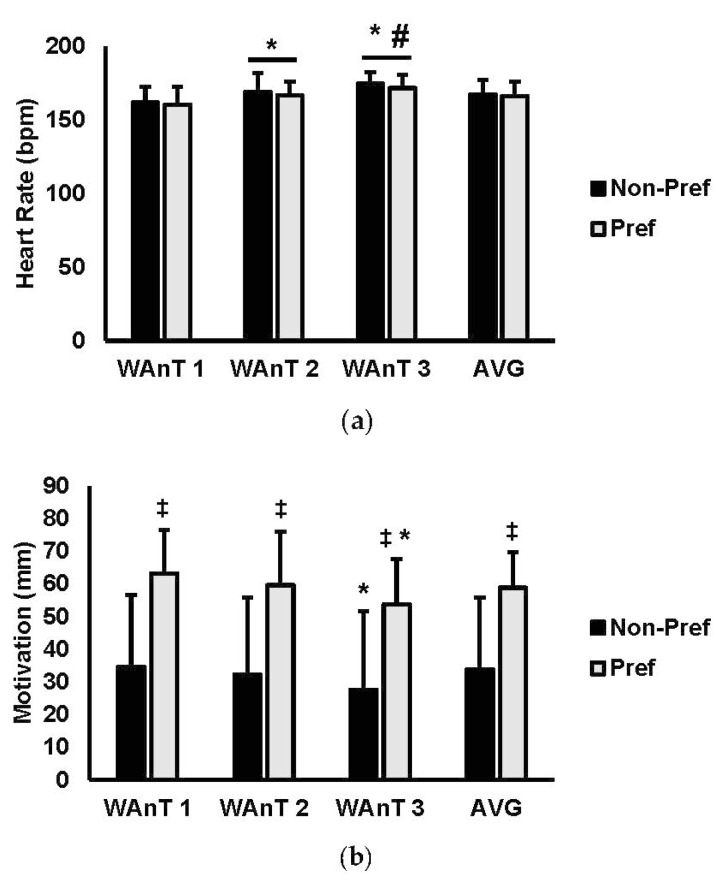

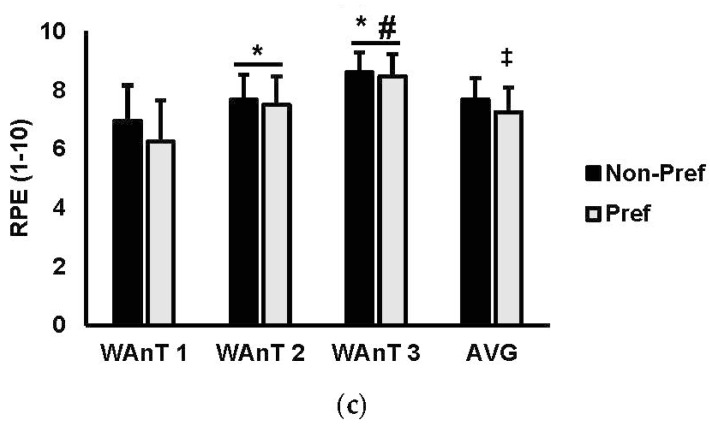
(**a**) Heart rate (bpm), (**b**) motivation (mm), (**c**) rate of perceived exertion (RPE) (1–10) for WAnT1, WAnT2, WAnT3, and the average (AVG) over all three. Data are presented as mean ± SD. * indicates significantly different from WAnT1. # indicates significantly different from WAnT2. ‡ indicates significantly different from non-preferred (NON-PREF) music.

Physiological and subjective outcomes are presented in Figure 2. For heart rate (bpm) (Figure 2a), there was a main effect for test (*p* < 0.001; η_p_^2^ = 0.762), while there was no main effect for condition (*p* = 0.576; η_p_^2^ = 0.012) or interaction for condition and test (*p* = 0.961; (η_p_^2^) = 0.003). Analysis of multiple comparisons for test revealed that the first test (PREF = 160.5 bpm ± 11.9, NON-PREF = 162.0 bpm ± 10.6) was significantly lower than both second (PREF = 166.7 bpm ± 9.3, NON-PREF = 169.0 bpm ± 12.2; *p* = 0.002) and third WAnTs (PREF = 171.7 bpm ± 9.0, NON-PREF = 173.9 watts ± 8.1; *p* < 0.001). In addition, the heart rate in the third WAnT was significantly higher than the second WAnT (*p* = 0.006). Average heart rate was not significantly different between music conditions (PREF = 166.3 bpm ± 9.9, NON-PREF = 167.5 bpm ± 9.8; *p* = 0.608; ES = 0.125).

For motivation (mm) (Figure 2b), there was a main effect for test (*p* = 0.006; η_p_^2^ = 0.337) and for condition (*p* < 0.001; η_p_^2^ = 0.385), with no interaction for condition and test (*p* = 0.961; η_p_^2^ = 0.012). Analysis of multiple comparisons for test revealed that the first test (PREF = 63.2 mm ± 13.2, NON-PREF = 34.5 mm ± 22.1) was significantly higher than the third WAnT (PREF = 53.7 mm ± 13.8, NON-PREF= 27.6 mm ± 23.9; *p* = 0.004). Average motivation was significantly higher for the preferred condition versus the non-preferred (PREF = 55.8 mm ± 10.8, NON-PREF = 33.9 mm ± 21.9; *p* < 0.001; ES = 1.520). 

For rate of perceived exertion (RPE; 1–10) (Figure 2c), there was a main effect for test (*p* < 0.001; η_p_^2^ = 0.774) while there was no main effect for condition (*p* = 0.374; η_p_^2^ = 0.041) or interaction for condition and test (*p* = 0.167; η_p_^2^ = 0.066). Analysis of multiple comparisons for test revealed that the first test (PREF = 6.2 ± 1.3, NON-PREF = 6.9 ± 1.2) was significantly lower than both second (PREF = 7.5 ± 0.9, NON-PREF = 7.6 ± 0.8; *p* < 0.001) and third WAnTs (PREF = 8.4 ± 0.7, NON-PREF = 8.6 ± 0.6; *p* < 0.001). In addition, the RPE in the third WAnT was significantly higher than the second WAnT (*p* < 0.001). Average RPE was significantly lower in the preferred music condition versus the non-preferred (PREF = 7.2 ± 0.8, NON-PREF = 7.7 ± 0.7; *p* = 0.028; ES = 0.540).

## 4. Discussion

Previous research has investigated the effects of listening to preferred versus non-preferred music on endurance and resistance exercise performance [1,2]. However, no studies have been conducted on music preference and repeated anaerobic cycling sprint ability. Thus, this investigation sought to describe the effects of listening to preferred versus non-preferred music on anaerobic cycling sprint performance using repeated WAnTs. These findings reveal that listening to preferred music does not impart ergogenic benefit versus listening to non-preferred music during repeated anaerobic cycling sprint exercise. However, preferred music increased motivation to exercise and decreased RPE. While the lack of ergogenic benefit from preferred music is in contrast with previous findings using other modes of exercise, the current findings may hold important implications for using music to endure repeated bouts of high-intensity sprint exercise. 

The current findings of no change in anaerobic performance are supported by other previous literature comparing music to no-music conditions [8,11]. Pujol et al. found no changes in power output or performance during 3 × 30 s WAnTs when comparing music to no music [11]. Taken together with our lack of increase in performance during 3 × 15 s WAnTs, music may not impart ergogenic benefit during high-intensity repeated sprints regardless of preference. One potential explanation for why no performance improvements were observed could be due to the intrinsic tempo of the music. For the current study, both the preferred and non-preferred songs were on average >120 bpm. Previous evidence has suggested that any song with a tempo >120 bpm can be considered stimulating and may enhance exercise performance [16,22]. It is possible that both the preferred and non-preferred conditions were stimulating for the participants thus, making them perform similarly. However, this is not fully supported as Atan et al. found no changes in power output using fast and slow tempo music during a single 30 s WAnT. How music preference and tempo interact to affect the ergogenic benefit of music is unclear and warrants further study. Our finding of no improvements in performance while listening to preferred music is in stark contrast to previous literature using different modes of exercise [1,2]. However, differences may be due to music synchrony and the pacing of exercise. Both Ballmann et al. and Nakamura et al. used bench press and endurance cycling for their modes of exercise. Due to the nature of the exercise modes, synchronization to pacing and rhythm of music may have occurred that could influence performance. This is supported by previous findings that synchronizing movements to music increases exercise efficiency during cycling [23]. Due to the maximal sprinting nature of WAnTs, participants may not have benefited from a pacing effect, which may have caused a lack of change in performance. This is supported by Simpson et al., that showed that synchronous music provides no additional benefit during 400 m sprints when compared to oudeterous music [12].

One of the main mechanisms by which music imparts ergogenic benefit is through disassociation and lowering RPE [4,5]. Potteiger et al. showed music decreased peripheral, central, and overall RPE when compared to a no-music condition [4]. In the current study, listening to preferred music caused a moderate-sized effect in decreasing average overall RPE. This result is supported by a previous investigation on preferred and non-preferred music where RPE was decreased during endurance cycling [2]. However, unlike the present study, RPE decreases occurred with increased performance. Discrepancies in the results may be due to the previously mentioned differences in the nature of the exercise bout, but also, differences in tempo of music in that their songs on average were <120 bpm and the current study was >120 bpm. Furthermore, the current investigation was standardized for volume, while their participants self-selected the volume. Other studies have also reported that participants felt exercise was easier with music with no accompanying physiological benefit [24]. Although no changes in performance were seen with changes in RPE, the observed decreases in RPE still may have important implications for enduring high-intensity sprinting. Since RPE decreased during the preferred music condition, individuals listening to preferred music may be able to exercise longer as a result. Pires et al. showed that RPE is a predictor for time to exhaustion at varying exercise intensities [25]. Further supporting this, Crewe et al. showed RPE as a predictor of exercise duration in a variety of environmental conditions [26]. However, ability to sustain exercise was not measured and future investigation is needed to determine if listening to preferred music allows for greater tolerance of exercise.

Another important mechanism by which music aids in boosting exercise performance is by increasing motivation. In the current study, preferred music had a large effect on motivation to exercise when compared to non-preferred music. This is in agreement with multiple previous investigations that music increases motivation during different modes of exercise. Hutchinson et al. showed that asynchronous music increased motivation and supramaximal cycling performance when compared to no music [10]. Supporting the use of preferred music, Ballmann et al. showed that motivation and resistance exercise performance was higher during preferred music conditions versus non-preferred [1]. It is important to note that preferred music had a large effect in their study, similar to the present investigation. In contrast, Ballmann et al. saw accompanying increases in performance with motivation. As previously mentioned, differences between the findings may be due to synchronizing music to the pace of exercise, but they also may be due to differences in genres picked by the participants. In their study, almost all of the participants picked the same genres for their preferred and non-preferred music, while the current study was much more heterogeneous in terms of genre selection. Of particular note, previous investigations have shown that whether a song is stimulating or not may rely on the genre rather than mere preference [27]. However, there have been other investigations which have shown increases in motivation without changes in exercise performance, which supports the current findings [28,29]. While increased motivation did not concurrently occur with changes in performance, these results hold important implications for increasing physical capacity. Previous evidence has shown that motivation has the ability to increase exercise capacity even without changing maximal physiological variables [30]. Thus, much like the observed decreases in RPE, increases in motivation did not change anaerobic performance but may play an important role in sustaining repeated bouts of high-intensity sprints. 

While the present investigation reveals novel information regarding music preference and anaerobic sprint performance, there were several limitations to the study. Only physically active young males were used and not females or trained anaerobic sprinters. Previous evidence has suggested that listening to music during anaerobic sprinting may affect long-distance runners and sprinters differently [31]. Thus, we cannot rule out that these results may be different depending on training status and population. Also, a no-music condition was not included in addition to preferred and non-preferred conditions. Multiple groups have investigated listening to music compared to no music during anaerobic exercise [8,9,11]. However, only conclusions comparing preferred to non-preferred music can be made using the current data set, leaving how these conditions compare to no music unknown. Lastly, it is customary to give participants verbal encouragement during WAnTs as it has been shown to positively affect performance [32]. Given that participants were focusing on the music they were listening to rather than verbal encouragement, our results may not be comparable to previous investigations utilizing verbal encouragement. In conclusion, preferred music appears to influence subjective measures such as RPE and motivation rather than performance. Future investigations should examine different populations and how music preference influences exercise tolerance and volume.
